# Alterations in the Gut Microbiota and Their Metabolites in Colorectal Cancer: Recent Progress and Future Prospects

**DOI:** 10.3389/fonc.2022.841552

**Published:** 2022-02-11

**Authors:** Jing Li, Ai-hua Zhang, Fang-fang Wu, Xi-jun Wang

**Affiliations:** ^1^ National Engineering Laboratory for the Development of Southwestern Endangered Medicinal Materials, Guangxi Botanical Garden of Medicinal Plant, Nanning, China; ^2^ National Chinmedomics Research Center, National Traditional Chinese Medicine (TCM) Key Laboratory of Serum Pharmacochemistry, Functional Metabolomics Laboratory, Department of Pharmaceutical Analysis, Heilongjiang University of Chinese Medicine, Harbin, China; ^3^ State Key Laboratory of Quality Research in Chinese Medicine, Macau University of Science and Technology, Macao, Macao SAR, China

**Keywords:** gut microbiota, metabolites, metabolomics, association analysis, colorectal cancer

## Abstract

Colorectal cancer (CRC) is a leading cause of cancer morbidity and mortality worldwide. The etiology and pathogenesis of CRC remain unclear. A growing body of evidence suggests dysbiosis of gut bacteria can contribute to the occurrence and development of CRC by generating harmful metabolites and changing host physiological processes. Metabolomics, a systems biology method, will systematically study the changes in metabolites in the physiological processes of the body, eventually playing a significant role in the detection of metabolic biomarkers and improving disease diagnosis and treatment. Metabolomics, in particular, has been highly beneficial in tracking microbially derived metabolites, which has substantially advanced our comprehension of host-microbiota metabolic interactions in CRC. This paper has briefly compiled recent research progress of the alterations of intestinal flora and its metabolites associated with CRC and the application of association analysis of metabolomics and gut microbiome in the diagnosis, prevention, and treatment of CRC; furthermore, we discuss the prospects for the problems and development direction of this association analysis in the study of CRC. Gut microbiota and their metabolites influence the progression and causation of CRC, and the association analysis of metabolomics and gut microbiome will provide novel strategies for the prevention, diagnosis, and therapy of CRC.

## Introduction

Colorectal cancer (CRC) is defined as a malignant tumor derived from intestinal epithelial cells that have characteristics of uncontrolled proliferation of cells, invasive nature, and metastasis. In 2020, there were 1.93 million new cancer cases worldwide, of which CRC incidence and mortality rate account for approximately 10% and 9.4%, respectively (1). As the third most common cancer worldwide, CRC has shown an increase in morbidity and mortality in younger individuals (2, 3). The majority of CRC is caused by pre-cancerous polyps, which are classified as either classic tubular adenomas or serrated polyps (4). Early detection and subsequent colonoscopic polypectomy (or surgery for malignant lesions) have improved significantly survival rates in recent years, but CRC screening rates in the general population remain relatively low due to a lack of distinct clinical symptoms and reliable screening approaches (5, 6), and information on participation and diagnostic yield of population-based CRC screening in China are limited (7). Thus, there is an urgent requirement to actively elucidate the pathogenesis of CRC and identify effective screening markers.

CRC development and progression may be linked to inheritance, immunity, environment, dietary habits, and lifestyle, nevertheless, the potential mechanism behind CRC remains unknown. Changes in the intestinal microbiota have been linked to CRC, according to recent studies (8). It has also been proposed that CRC is fundamentally a genetic as well as a microbiological disease (9). Emerging studies have suggested that certain pathogens and/or microbial communities play a significant role in tumorigenesis by activating inflammatory pathways and aberrant epithelial cell proliferation, boosting tumorigenic immune responses, inducing DNA damage, and altering genome stability (10). Moreover, dysbiosis of the resident gut microbiota (rather than simply certain pathogens), particularly their metabolites, has been shown to significantly alter cancer risk or progression by causing immune response abnormalities or others (10–12). Furthermore, the reduction of several beneficial gut microflora metabolites, such as short-chain fatty acids (SCFAs), plays an important role in tumorigenesis and development.

Research on microbe-derived metabolites has significantly expedited our comprehension of the host-microbiota metabolic interactions in CRC (13), thanks to the constant development and improvement of metabolomics technology (14). The integration of functional prediction based on metagenome sequencing and characterization of microbial metabolites based on metabolomics can provide unique insights into the relationship between intestinal microbiota imbalance and the production of harmful metabolites that cause colorectal carcinogenesis (15). In recent years, a better comprehension of the characteristics of intestinal flora and the development of metabolomics technology have provided new insights into CRC integration research, mainly including a deeper understanding of the pathogenesis of CRC (16, 17), the search for non-invasive early period diagnosis and disease recurrence prediction markers (18–20), the identification of new therapeutic targets and drug treatment mechanisms (21), and so on. This review summarizes recent advancements in research on the alterations of intestinal flora and its metabolites associated with CRC, association analysis of metabolomics and gut microbiome in the diagnosis, prevention, and therapies of CRC, and discuss the current challenges and future research directions on a scientific basis.

## Alterations of Gut Microbiome Associated With CRC

The gut microbiota is the human body’s biggest symbiotic ecosystem, with more than10^13^ microorganisms (22). Bacteria, fungi, and viruses constitute the collection of microorganisms residing within the gastrointestinal tract, with bacteria accounting for the greater proportion (23). The human gut microbiota is critical to human health by forming a symbiotic relationship of mutual advantages, interdependence, and mutual restrictions with the host. The key elements that determine the composition and activity of intestinal microbiota dysbiosis include age, diet, medicines, and lifestyle (24–26). Recent research discovered that host gene mutations are closely associated with gut microbiome dysbiosis (27). Intestinal flora imbalance reduces the function of the intestinal mucosal barrier and enhances bacterial translocation, resulting in inflammation and infection (28). A few studies have suggested that gut microbiota disorders are linked to many diseases, including inflammatory bowel disease, neurological diseases, metabolic syndrome, heart disease, diabetes, and several malignancies (29–34). As a result, the intestinal microbiota is thought to be a potential therapeutic target for various illness interventions.

Multiple studies have shown that gut microbes play a role in CRC carcinogenesis. The gut microbiota was first implicated in CRC formation in germ-free rats in the 1970s, and the intestinal microflora affects the carcinogenic and/or cocarcinogenic action of several compounds in the large intestine (35). Another study discovered that the fecal flora of CRC patients can induce carcinogenesis in germ-free mice and conventional mice exposed to a carcinogen (36). Intestinal microbial dysbiosis was detected in animals with both spontaneous and chemically induced colon cancer. For example, the azoxymethane/dextran sodium sulfate-induced CRC mice suffered from intestinal flora alteration (37). Apc^min/+^ is a mouse model of spontaneous intestinal polyposis that closely resembles familial adenomatous polyposis in humans. One study found that CRC patients’ gut microbiota accelerated the growth of intestinal adenoma in Apc^min/+^ mice (38).

### The Interaction Between Environmental Factors and Gut Microbiome

Environmental exposures such as diet, nutrition, and lifestyle have all been postulated to influence the development or progression of CRC, presumably *via* the complex metabolic and immunological pathways. The gut microbiota has been demonstrated to play a crucial role in the initiation and progression of CRC as an important metabolic and immunological regulator. Increasing evidence suggests that environmental factors are important determining factors of gut microbial shape community composition and function and that changes in these factors cause changes in host gene expression, metabolic function, and local and systemic immune response, all of which affect tumor progression.

A study found an elevated risk of CRC in persons who ate a western-style diet that is rich in red and processed meats, alcohol, and low in fiber from bread and morning cereals, but not in people who ate fish, poultry, cheese, fruit, vegetables, tea, or coffee (39). The consumption of red and processed meats alters the gut microbiome’s stability, perhaps increasing the production of multi-site carcinogens such as N-nitroso compounds, heterocyclic amines (HCAs), polycyclic aromatic hydrocarbons (PAHs), and trimethylamine-N-oxide (TMAO) (40). Excessive consumption of red and processed meats will promote the proliferation of N-nitroso-producing bacteria such as facultative and anaerobic colonic bacteria, explaining the epidemiologic link between red meat consumption and CRC (11). Exposure to HCAs and PAHs has been related to alterations in the abundance and composition of gut bacteria, as well as moderate inflammation in the ileal and colonic mucosa (40). Whereas gut microbiota could directly increase the bioactivation and transformation of PAH and HCA into estrogenic metabolites and HCA-M1, hence decreasing the carcinogenic risk of HCA and PAH (41). TMAO is a metabolite that is mostly controlled by the composition or structure of the gut microbiota, and it can cause an inflammatory response and contribute to colon carcinogenesis (42). Another study discovered that *Eubacterium limosum* has the capacity to reduce TMAO levels in the gut (43). Long-term dietary treatment with fiber-rich foods increases the abundance of butyrate-producing bacteria such as *Prevotella*, but adherence to low-fiber diets increases the abundance of *Bacteroides*, which has been linked to CRC (44). Several studies have linked a high-fat diet for an extended period to a change in microbial communities defined by a rise in Firmicutes and the phylum Actinobacteria and a loss in Bacteroidetes, which alters bile acid metabolism. Bile acid, in turn, can influence the composition of gut bacteria (45). These remarkable findings show that diet plays a vital role in influencing gut flora and preserving colonic health.

Exercise restores bacterial homeostasis by boosting the relative number of butyrate-producing bacteria and the ratio of Bacteroidetes to Firmicutes, which can enhance the intestinal level of butyrate and short-chain fatty acids (SCFAs) and lower the risk of CRC (46). On the contrary, smoking-related microbial alterations have been linked with a higher risk of CRC with a longer latency period (46). The precise processes by which each environmental factor may influence CRC differ, nonetheless, the interaction between environmental variables and gut microbiome may impact colorectal tumorigenesis *via* changes in the host metabolism and immune system.

### The Link Between Genetic Mutations and Different Gut Microbiome Profiles

Based on the origin of the mutation, CRC caused by mutations can be classified as sporadic (70%), inherited (5%), or familial (25%). The carcinogenic mechanism by which mutations induce CRC is mainly classified into three categories: chromosomal instability (CIN), microsatellite instability (MSI), and the CpG island methylator phenotype (CIMP) (47). All of these potential mechanisms induce changes in DNA, RNA, or metabolites, which can serve as the potential predictive biopsy biomarkers. To better understand the CRC mechanism, further research included a more in-depth analysis of the link between mutations and gut microbiota. BRAF mutation is linked to DNA methylation in serrated polyps and CRC. A previous study has discovered that the BRAFV600E mutation causes a unique gut microbiome signature (*Prevotella enoeca* and *Ruthenibacterium lactatiformans*) (48). And, *Fusobacterium nucleatum* positive was significantly related to MSI-high status and CIMP (49). Some gut microbiome, such as *pks*^+^ *Escherichia coli*, enterotoxigenic *Bacteroides fragilis*, *Faecalibacterium prausnitzii*, and *Fusobacterium mortiferum*, may be able to predict the development of CRC from intestine adenomatous polyps (27, 50). Some sporadic CRC cases have mutations or those who have epigenetically silenced MMR genes, emphasizing the danger of DNA damage caused by pathogens and gut-associated microbes (51). Overall, these basic findings highlight the importance of some gut microbiota in inducing colorectal carcinogenesis and encourage further research into other putative gut microbiota that can lead to CRC development.

### The Mechanisms of Colon Carcinogenesis Mediated by Gut Microbiota

Thanks to high throughput sequencing technologies, we are learning more about the microbial environment in our gut microbiome, as well as the involvement of the gut microbiome in people colonization tumors and nontumor colonic locations (34, 52). When the makeup of bacterial species and the number of harmful bacteria change, microbiota dysbiosis occurs. Dysbiosis of the colon microbiota is the driving force behind colon carcinogenesis (10). The causal association between the presence of particular microorganisms and the development of CRC has also been verified. Previous research points to increased bacterial richness and diversity in CRC, as well as various variations in microbial community composition (53). Furthermore, a fresh study has revealed the significance of fungus in the development of colorectal tumors. According to recent research, CRC-associated mycobiome dysbiosis is characterized by changes in fungal composition and ecology (54, 55). Also, the quantities of several particular fungi are elevated in CRC patients and could be exploited as a potential diagnostic biomarker for adenomas (56). Viruses, in addition to bacteria and fungi, have appeared with identifiable disease signatures in CRC. Findings from Hannigan et al. (57)suggested the virome has an indirect impact on CRC *via* modifying the related bacterial ecology. **Table 1** lists the intestinal microbiota associated with CRC.

**Table 1:** Studies of gut bacteria associated with the development of adenoma and/or CRC.

Phyla	Microorganism	Variation in CRC	Effectors/metabolites	Related Mechanism	Ref.
Proteobacteria	*pks*^+^ *Escherichia coli*	↑	Colibactin	DNA damage	(58–61)
Fusobacteria	*Fusobacterium nucleatum*	↑	Adhesin FadA, Fap2/SCFAs, formyl methionyl leucyl phenylalanine	Wnt/β-catenin signaling	(62)
Bacteroidetes	Enterotoxigenic *Bacteroides fragilis*	↑	BFT	Wnt/β-catenin, MAPK, IL-17R, NF-κB, Stat3 signaling	(63, 64)
Firmicutes	*Enterococcus faecalis*	↑	ROS	Wnt/β-catenin, formation of bacterial biofilms, DNA damage	(65–68)
Firmicutes	*Streptococcus gallolyticus subsp. gallolyticus*	↑	Gallocin	Wnt/β-catenin	(69)
Firmicutes	*Peptostreptococcus anaerobius *	↑	TLR2 and TLR4	PI3K-Akt-NF-κB signaling	(70, 71)
Firmicutes	*Lactobacillus*	↓	lactic acid, bile acid hydrolase	Wnt/β-catenin, bile acid metabolism	(72, 73)
Actinobacteria	*Bifidobacterium*	↓	bile acid hydrolase	bile acid metabolism	(73–75)
Firmicutes	*Streptococcus thermophilus*	↓	β-galactosidase	Hippo signal, the Warburg effect	(76)
Firmicutes	*Butyricicoccus pullicaecorum*	↓	butyrate	SCFAs metabolism.	(77)
Firmicutes	*Clostridium butyricum*	↓	butyrate	SCFAs metabolism, Wnt/β-catenin	(78)
Firmicutes	*Faecalibaculum rodentium*	↓	SCFAs	inhibiting calcineurin/NFATc3 activation	(79)
Firmicutes	*Holdemanella biformis*	↓	SCFAs	inhibiting calcineurin/NFATc3 activation	(79)

Up arrow indicates increase, down arrow indicates decrease.

*Escherichia coli (E. coli)* is the most common aerobic gram-negative bacterium in the normal intestinal flora, and it is essential for promoting intestinal flora stability and maintaining normal intestinal homeostasis. The presence of *E. coli* strains to carry the genotoxic island *pks*^+^, which synthesizes the colibactin genotoxin (58). Colibactin causes DNA damage that increases the risk of CRC (59, 60, 80).

*Fusobacterium nucleatum* is a form of oral symbiotic bacteria that has been confirmed to be related to CRC over the past decade (81). *Fusobacterium nucleatum* has been discovered to play a function in impacting cancer cells or modulating the tumor microenvironment, influencing the progression, metastasis, and chemoresistance of CRC (82). The most important carcinogenic mechanism of *Fusobacterium nucleatum* is immune modulation, virulence factors, microRNAs, and bacteria metabolism (83). Recent research has found that *Fusobacterium nucleatum* promotes CRC by inducing Cdk5-activated Wnt/β-catenin modulator annexin A1 (62, 84). Also, high levels of *Fusobacterium nucleatum* can diminish NK cell function, and this decrease in NK cell activity may be associated with increases in proinflammatory cytokines (IL-1β and TNF-α) following *Fusobacterium nucleatum* therapy (85).

Enterotoxigenic *Bacteroides fragilis*, a common gram-negative anaerobe that produces *Bacteroides fragilis* toxin (BFT), induces inflammatory diarrhea and tumors associated with inflammation (86). The mucosal BFT exposure is prevalent and may be a contributing factor and screening marker for CRC development (87). The BFT initiates a pro-carcinogenic multi-step inflammatory cascade that requires Wnt/β-catenin, MAPK, IL-17R, NF-κB, Stat3 signaling, among other things (63, 64).

Although *Enterococcus faecalis* was previously thought to be a normal gram-positive bacterium of the gut microbiome, new evidence reveals that it is inherently connected to CRC (88). There has also been evidence of a link between enterococcal endocarditis and hidden CRC (89). *Enterococcus faecalis* was found to promote the proliferation of HCT116 colon cancer cells (90). This bacterium has also been shown to colonize the murine gastrointestinal tract by activating Wnt/β-catenin signaling (65), forming bacterial biofilms (66), promoting DNA damage, arresting the cell cycle, and inducing pluripotent transcription factors through increased reactive oxygen species (ROS) production (67, 68).

*Streptococcus gallolyticus subsp. Gallolyticus*(Sgg), originally known as *S. bovis* biotype I, is an opportunistic gram-positive pathogen. Sgg colonization occurs in the gut, which has been associated with the development of CRC (91, 92). Kumar R et al. (91) discovered that the particular cell environment, bacterial growth phase, and direct interaction between germs and CRC cells may boost Sgg colonization of the gut to outcompete commensal members and enhance colon cancer cell proliferation. Sgg produces gallocin, a bacteriocin that is increased by bile acids and may be harmful to commensal members (93). Furthermore, the significant activation of the Wnt signaling pathway and decreased levels of the bile acid apex transporter gene Slc10A2 can influence the formation of mutations in APC, which supports Sgg colonization in the gut (69). According to a recent study, SggT7SST05 is a previously unknown pathogenic factor that can induce Sgg to colonize the colon and promote CRC (94).

*Peptostreptococcus anaerobius*, a type of gram-positive anaerobic bacteria that normally reside in the mouth cavity and intestines, was shown to be considerably concentrated in the feces and tissues of CRC patients. The researchers discovered that *Peptostreptococcus anaerobius* anaerobic increases cholesterol production and cell proliferation in a ROS-dependent manner *via* acting on TLR2 and TLR4, consequently boosting the formation of colon cancers (70). Long et al. (71) showed that *Peptostreptococcus anaerobius* causes CRC through a PCWBR2-integrin α2/β1-PI3K-Akt-NF-κB signaling axis and this axis has been identified as a possible treatment target for CRC.

Some bacteria, as previously established, have a pathogenic function in CRC, whilst others play a preventive role. The most common type of probiotics is lactic acid bacteria, which appropriate doses are good for the health of the host. There is evidence that lactic acid bacteria, particularly *Lactobacillus* species, used clinically as a supplement for prevention and therapy of CRC reduced the onset or progression of the disease by altering the Wnt/β-catenin signaling pathway (72). The genus *Bifidobacterium* is one of the most common bacterial populations in the bowel and is found in every healthy human gut. Fahmy et al. (74) research showed that treating CRC mice with *Bifidobacterium longum* decreased NF-κB and IL-6 concentrations, increased IL-1β concentrations, reduced the number of aberrant crypt foci, and slowed CRC progression. As well, many *Bifidobacterium* species demonstrated anticancer action on CRC cells by decreasing and boosting anti-apoptotic and pro-apoptotic genes (75). Some *Lactobacillus* and *Bifidobacterium* species generate bile acid hydrolase (73), which participate in bile acid metabolism, thereby specifically affecting the development of CRC (95). Research shows that *Streptococcus thermophiles* suppressed cell proliferation, reduced colony formation, induced cell cycle arrest, and promoted apoptosis through β-Galactosidase produced by the bacterial community (76). The production of β-galactosidase results in the release of galactose, which suppresses the Hippo signal and alters the Warburg effect (76). According to certain studies, a wide range of bacteria that produce SCFAs regulates the SCFAs transporter, which slows the advancement of CRC. By activating the SCFAs transporter and/or receptor, the butyrate-producing bacterium *Butyricicoccus pullicaecorum*, for example, may enhance the clinical prognosis of CRC (77). *Clostridium butyricum*, a probiotic that produces butyrate, can suppress CRC development *via* regulating Wnt/β-catenin signaling and gut flora (78).

## Gut Microbiota-Derived Metabolites as Key Actors in CRC

Despite significant efforts and breakthroughs in comprehending the composition of the human gut flora, many functional features remain unknown. The ability of much intestinal flora to metabolize simple compounds results in various bioactive metabolites that interact with a wide range of receptors within the host. As a result, communication between diverse gut microorganisms and the host is primarily accomplished *via* the metabolic super-pathway (96). To comprehend this communication, we must first be able to characterize the huge number of metabolites produced by bacteria in reaction to their surroundings, as well as how the host reacts to these metabolites (96).

### Short-Chain Fatty Acids (SCFAs)

**Figure 1:**
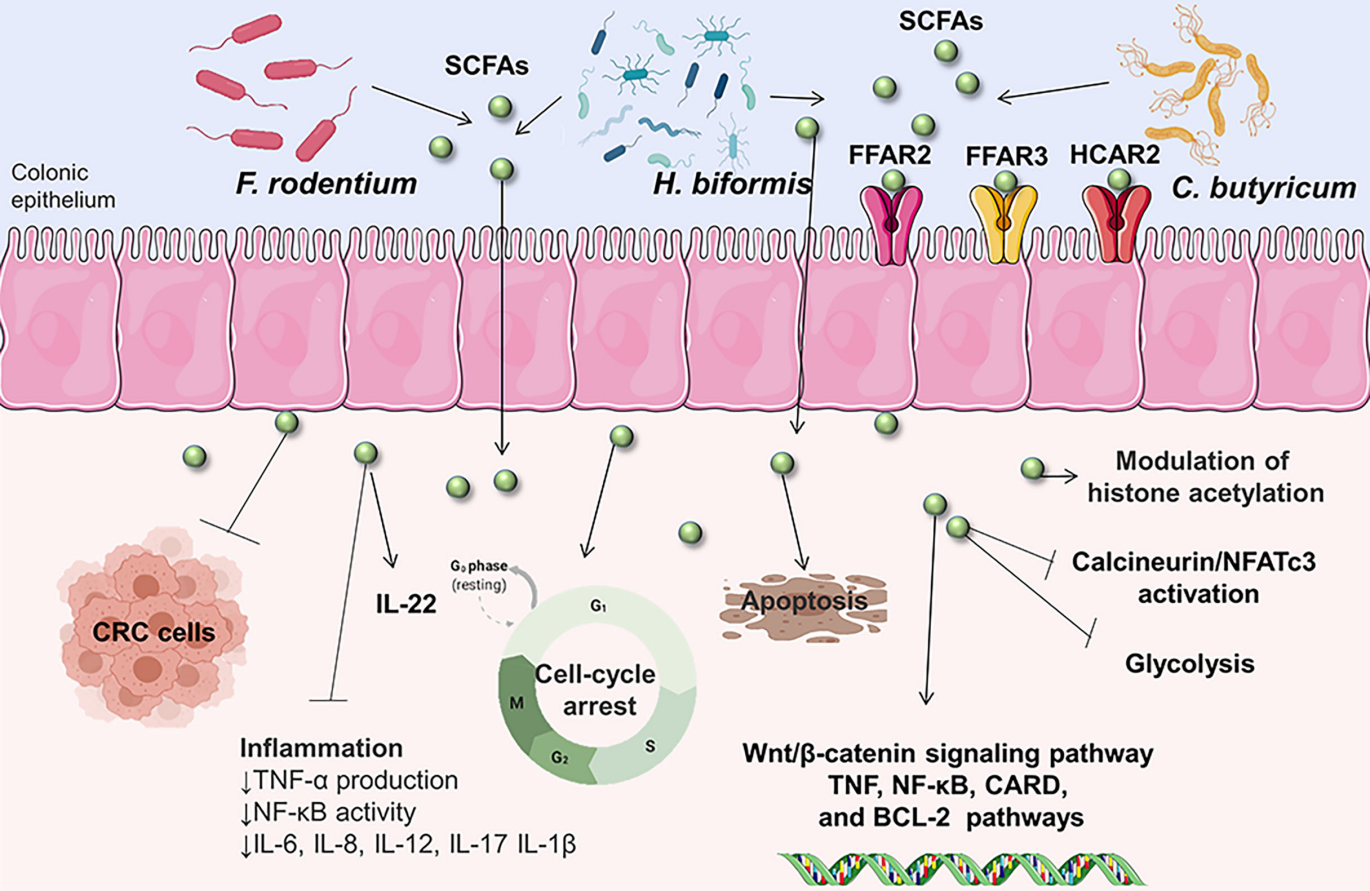
The schematic illustration of different cell processes triggered by SCFAs in CRC cells. Gut microbes catabolize unabsorbed dietary nutrients producing SCFAs. SCFAs can affect the inflammatory and immunological responses, colon cancer cell cycle arrest and apoptosis by influencing various receptors, signaling, apoptotic gene expression, which may be linked to the onset and progression of CRC. NFATc3, nuclear factor of activated T cells 3;CARD, proteins containing a caspase-associated recruitment domain; TNF, tumor necrosis factor; NF-κB, nuclear factor kappa-light-chain-enhancer of activated B cells; Bcl-2, B cell lymphoma protein-2; IL-6, interleukin-6; TNF-α, tumor necrosis factor-α; IL-22, interleukin-22; IL-8, interleukin-8; IL-12, interleukin-12; IL-17, interleukin-17; IL-1β, interleukin-1β.

SCFAs are predominantly acetic acid, propionic acid, and butyric acid, which are the most important microbial such as *Faecalibaculum rodentium* (*F. rodentium*), *Holdemanella biformis* (*H. biformis*), and *Clostridium butyricum* (*C. butyricum*) metabolites of dietary fiber. According to published reports, combined dosing of SCFAs inhibited tumor formation and reduced colon inflammation in a mouse model of CRC associated with colitis (97). SCFAs inhibit calcineurin/NFATc3 activation and thus contribute to control protein acetylation and tumor cell proliferation (79). SCFAs induce apoptotic cell death in CRC cells by pathways involving lysosomal membrane permeabilization, which is linked to mitochondrial malfunction and degradation (98). Moreover, *C. butyricum* can suppress the growth of intestinal tumors by regulating Wnt/β-catenin signaling, lowering proliferation, and promoting apoptosis (78). Also, a great promotive efficacy of SCFAs promotes human colon cancer cell cycle arrest and apoptosis *via* influencing apoptotic gene expression that is mainly involved in the TNF, NF-κB, CARD, and Bcl-2 regulated pathways (99). SCFAs can activate the free G protein-coupled receptors fatty acid receptor 2 (FFAR2), FFAR3, and hydroxycarboxylic acid receptor 2 (HCAR2) receptors on intestinal epithelial cells and immune cells, triggering a cascade of inflammatory and immunological responses that help to tissue integrity and host defense (100–102). In addition, the interaction between SCFAs transporters and glycolysis may be linked to the onset and progression of CRC (103) (**Figure 1**).

### Bile Acids (BAs)

**Figure 2:**
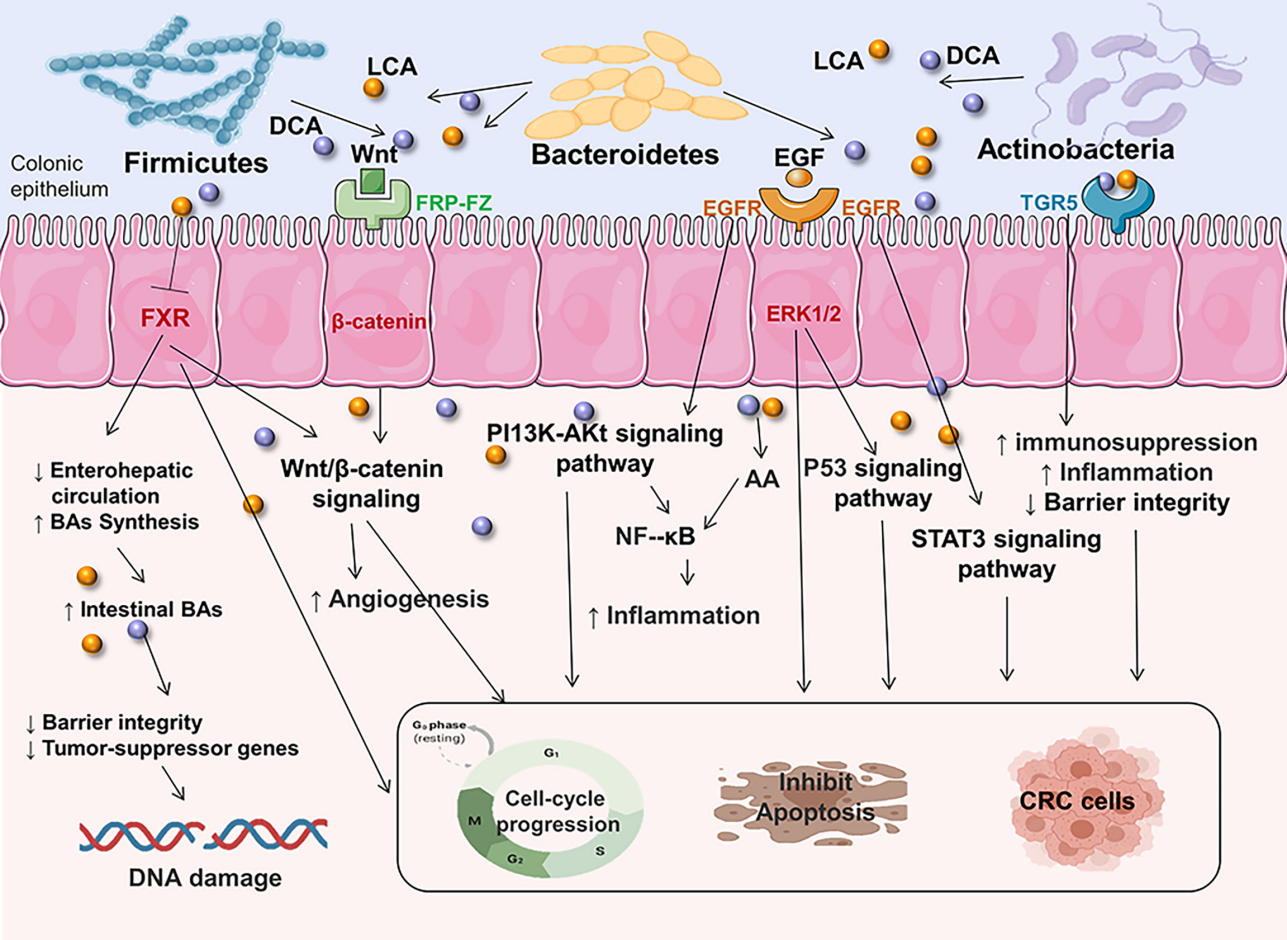
Secondary BAs promote CRC initiation and progression by inducing CRC-associated signaling. DCA and LCA are major secondary bile acids produced by gut bacteria through cholic acid metabolism, which can bind to host receptors, including nuclear hormone receptor FXR and G-protein-coupled receptor 1 (TGR5). This can increase the risk of CRC by triggering multiple cellular signals and genotoxicity, disrupting epithelial barrier integrity, driving inflammation immunological dysfunction. Multiple signaling pathways are involved in complex disease process. EGFR, epidermal growth factor receptor; FXR, farnesoid X receptor; Wnt, wingless-related integration site; ERK1/2, extracellular signal-regulated kinase 1/2; AA, arachidonic acid; PI3K/AKT, phosphatidylinositol-4,5-bisphosphate 3-kinase/serine-threonine kinase; EGF, epidermal growth factor; STAT3, signal transducer and activator of transcription 3.

BAs are formed from cholesterol in the liver as primary bile acids and are then released into the gut, where they are further metabolized by specific gut microorganisms [three major phyla: Firmicutes, Bacteroidetes, and Actinobacteria (73)] to genotoxic and proinflammatory secondary BAs (104). High-fat diets increase colonic excretion of secondary bile acids, particularly deoxycholic acid (DCA) and lithocholic acid (LCA), which leads to decreased activation of functional farnesoid X receptor (FXR) signaling in CRC cells, promoting colonic carcinogenesis and CRC risk (45, 105, 106). Among nuclear receptors, FXR has a tumor-suppressive action that can prevent the beginning of CRC by modulating FXR-regulated transcriptional and epigenetic processes in intestinal cancer stem cells (105, 107, 108). Moreover, recent research has indicated that FXR deficiency not only impairs enterohepatic circulation and bile acid production, but also enhances Wnt/β-catenin signaling, which promotes DNA damage, tumor development, and the prevention of apoptosis (105, 109). Also, the absence of FXR causes genotoxic activity and disrupts epithelial barrier integrity leading to tumor promotion (45). In addition, bile acids are also G-protein-coupled receptor 1 (TGR5) ligands on the cell surface, which regulate intestinal barrier formation and inflammation-driven immunological dysfunction (110, 111), both of which are linked to the development of CRC. The EGFR pathway has also long been linked to the progression of CRC. The binding of a ligand like EGF to EGFR stimulates the stimulation of downstream signaling cascades such PI3K/AKT, P53, and STAT3 signaling pathways, which are linked to tumor cell proliferation, survival, angiogenesis, invasion, and metastasis (112–115). NF-κB is also one of the key signaling pathways triggered by increased AA release as a result of prolonged intestinal exposure to secondary BAs (116). This signaling is also induced as a downstream effect of PI3K/AKT signaling to promote inflammation in the intestine (117). The increased intestinal inflammatory state later leads to dysbiosis and raises the chance of developing CRC (**Figure 2**).

### Tryptophan (Trp) Metabolites

**Figure 3:**
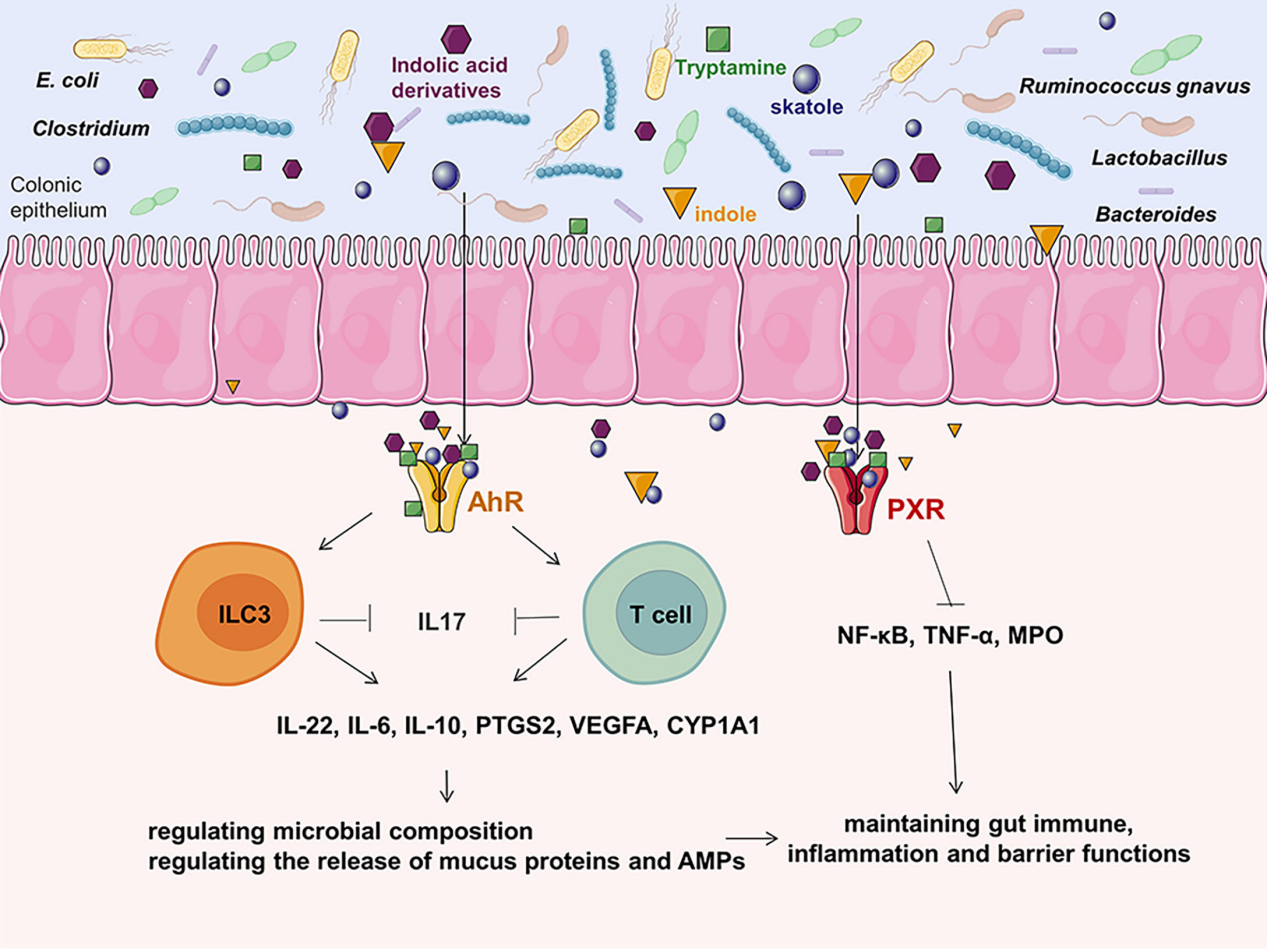
The effects of Trp metabolites on CRC.As one of the most potent bioactive metabolites, bacterial Trp metabolites can activate the cytosolic ligand-activated transcription factor AhR and PXR, which can influence the release of cytokines and modulate gut immunological, inflammation, and barrier functions. ILC3, innate lymphoid cell 3; VEGFA, vascular endothelial growth factor A; MPO, mucosal myeloperoxidase; AMP, antimicrobial peptides; PTGS2, prostaglandin G/H synthase 2; PXR, pregnane X receptor; CYP1A1, cytochrome P450 1A1; AhR, aryl hydrocarbon receptor.

Trp can be directly utilized by *E. coli*, Firmicutes *Clostridium sporogenes*, *Ruminococcus gnavus*, *Lactobacillus*, *Clostridium*, *Bacteroides*, and others to produce indole, indican, tryptamine, and skatole as well as indole acid derivatives (118). Increased Trp metabolism has been linked to the occurrence of CRC and inflammatory bowel disease (119, 120). Previous research has found a decreased indole to Trp ratio in CRC patients when compared to healthy controls (121). This change in the indolic pathway may result in an increased inflammatory response in colon carcinogenesis, impacting aryl hydrocarbon receptor (AhR) signaling (118). The AhR activated by bacterial Trp metabolites releasing the secretion of cytokines like IL-22, IL-6, IL10, PTGS2, VEGFA, CYP1A1 and reducing pro-inflammatory cytokines like IL-17 could regulate the release of mucus proteins such as Mucin 2 and antimicrobial peptides in intestinal epithelial cells so that maintain gut immune, inflammation and barrier functions (118, 119, 121–124). Furthermore, bacterial Trp metabolites are also ligands for PXR. The PXR induced by Trp metabolites suppresses the action of MPO and pro-inflammatory cytokines such as NF-κB, TNF-α, which can modulate gut immunological, inflammation, and barrier functions, hence reducing the development of CRC (125) (**Figure 3**).

In addition to the main metabolites discussed above, hydrogen sulfide (H2S), lactate, succinate, trimethylamine-N-oxide, N-nitroso compounds, and bacterial toxin also contributed to colon carcinogenesis partly through its proinflammatory property or the angiogenic effect or stimulating immune responses or others (126).

## Metabolomics Discloses CRC Biomarkers Associated With Gut Flora

Despite being the gold standard for detecting and removing premalignant colorectal lesions (127), colonoscopy has drawbacks such as high prices, the risk of complications (128–130), the limited capacity of medical centers, and patient discomfort due to its intrusive nature (131). The public’s interest has been aroused by non-invasive CRC screening technologies including the Guaiac fecal occult blood test (gFOBT) and fecal immunochemical test (FIT) (132). However, the usefulness of these non-invasive approaches for CRC screening is still restricted by hemoglobin degradation and intermittent bleeding patterns, resulting in a significant number of CRC cases being detected late, leading to a poor prognosis (133, 134). To develop innovative approaches for CRC early diagnosis and screening, many biomarkers have been explored that are detectable in non-invasively acquired samples of feces, colon mucus, blood, urine, saliva, and exhaled air. Although promising outcomes are frequently reported, striking the correct balance between technological complexity, cost, and diagnostic efficacy of novel approaches is difficult (135). To further lower the prevalence of CRC and accompanying mortality rates, it is urgently needed to maximize detection accuracy using a cost-effective non-invasive CRC screening technique.

With the rapid advancement of next-generation sequencing technology, metagenomic sequencing now presents a formidable platform for study into intestinal flora (136, 137). One significant goal of metagenomics is to use DNA sequence data to analyze the frequency of taxa and gene functions within natural microbial communities such as viruses, bacteria, and fungi (138). Metagenomics has the benefit of not needing the cultivation of individual species or prior sequence information to known genes (139), making it a useful way for gaining a more in-depth comprehension of the intestinal bacteria and molecular pathogenesis of CRC, as well as establishing targets for new therapeutic strategies. However, the technology has significant limitations and cannot be utilized to better understand the function of intestinal bacteria by relying solely on DNA analysis. Metabolomics, on the other hand, is a promising method of encouraging functional research on the gut microbiome (140–142). Using the new paradigms of systems biology and pathophysiology to investigate specific microbial-associated metabolites that could be employed as biomarkers for illness diagnosis and treatment.

Metabolomics, as a part of systems biology, has emerged as a fresh study method for the post-genomic era in recent years (143–145). Metabolomics employs many analytical techniques to detect, identify, and quantify a wide range of overall and dynamic changes in endogenous metabolites in biological samples as a result of disease onset and intervention (146). Metabolomics can be used to discover dynamic changes within or across groups by integrating high-throughput sequencing technology with univariate and multivariate statistical techniques, allowing for a more holistic look at pathogenic and therapeutic causes (147–149). Metabolomics analysis has been shown to be suitable for investigating quantitative measurements of microbiome-derived or microbiome-modified metabolites, providing a functional read-out of microbiota metabolic activities and host-microbiome interactions (150). This method is an effective tool for identifying and validating microbial community-based biomarkers.

Chen F et al. (151) had used comprehensive analysis of untargeted/targeted serum metabolomics and metagenome sequencing of paired fecal samples to develop a model based on changes in gut microbiome-associated serum metabolites (GMSM) that can distinguish patients with CRC and adenoma from healthy normal individuals better than the clinical marker carcinoembryonic antigen. Similarly, Clos-Garcia et al. (19) used an integration of metabolomics and microbiome data analysis to find possible biomarkers for both advanced adenomas (AD) and CRC from feces samples. They discovered variations in the quantities of cholesterol esters and sphingolipids in the stool of CRC patients. Also, *Fusobacterium*, *Parvimonas*, and *Staphylococcus* increased in CRC patients while the *Lachnospiraceae* family decreased. *Adlercreutzia* is more common in the stool of AD patients. This work discovers potential early biomarkers that exceed existing diagnostic methods and contextualizes them within the gut microbiota’s proven role in CRC etiology. Yang Y et al. (18) used 16S rRNA gene sequencing and gas chromatography-mass spectrometry (GC-MS) to analyze the microbiome and metabolome of fecal samples taken from CRC patients and healthy participants. Their findings highlighted an enrichment of metabolites (i.e. polyamines) as a result of the CRC-associated fecal microbiota imbalance. Tang Q et al. (152) investigated the progression of ulcerative colitis (UC) into CRC in rats based on the connections between the gut microbiome and the metabolic profiles in the body through ultra-high-performance liquid chromatography and electrospray ionization quadrupole time-of-flight tandem mass spectrometry (UPLC-Q-TOF-MS/MS) metabolomics and 16S rDNA sequencing technology. The findings suggest that linoleic acid and 12−hydroxy−8,10-octadecadienoic acid could be important biomarkers for CRC progression in individuals with ulcerative colitis when paired with Enterobacteriaceae and Proteobacteria enrichment. The above results show that metabolomics applies to the analysis of microbe-associated metabolites that has the potential to be employed as CRC diagnostic biomarkers in therapeutic explorations.

## The Application of Association Analysis of Metabolomics and Gut Microbiome

The fact that CRC pathogenesis has been extensively researched for many years. CRC is still difficult to treat, and the majority of patients will die as a result of the condition (153). As a result, innovative anti-cancer medications that are effective or improve on existing treatments are critically needed. Cytotoxic chemotherapeutics, which work by destroying rapidly reproducing cancer cells, are still one of the most preferred techniques for treating various tumors (154). However, due to side effects such as bone marrow injury and gastrointestinal toxicity, which can result in myelosuppression and diarrhea, the usage of this medicine is restricted. Therefore, effective antitumor medicines may be required to provide maximum antitumor impact with minimal side effects, which has emerged as a research hotspot and challenge in the field of cancer research. The symbiotic microbe-host interactions may have an impact on both the efficacy and toxicity of anticancer medicines. The possible function of gut flora in cancer prevention and treatment has received a lot of attention (155). How interactions of anticancer drugs with microbiome and metabolome affects cancer development and treatment is considered one of the research frontiers in the fight against CRC (156–158). As we all know, the microbiome cannot directly determine the creation of microbial metabolic products. The influence of a microbial interaction network and dynamic changes in microbial metabolites on CRC pathogenesis issues cannot be predicted by studying the involvement of a single microorganism in CRC pathogenesis (12, 15). Thus, the potential significance of intestinal microbial metabolites in the etiology of CRC must be evaluated within the framework of metabolomics and gut microbiome association analysis, which is important in terms of the development of new strategies and medications to prevent and treat CRC.

Ji et al. (159, 160) found that an active polysaccharide (ZMP) purified from jujube fruit significantly reduced the Firmicutes/Bacteroidetes abundance and pro-inflammatory cytokines, increased the richness of *Bifidobacterium*, *Bacteroides*, *Lactobacillus*, and the concentration of SCFAs, and was effective in preventing and treating DSS/AOM-induced CRC in a mouse model by analyzing fecal-microbiota composition and fecal-metabolome profiles. In addition, there is a strong relationship between the fluctuant gut microbiota and the metabolites. These findings shed light on the mechanisms behind the impact of dietary ZMP on host health. It has also been reported that oral administration of American ginseng significantly reduced AOM/DSS-induced colitis and colon carcinogenesis *via* reducing cytokines (IL-1α, IL-1β, IL-6, G-CSF, and GM-CSF) production and restoring the profiles of the plasma and stool metabolomics and microbiota, especially upregulating the metabolites of glutamine, aminomalonic acid, 6-P-glucose, and others and the expression of Firmicutes while downregulating *Bacteroidales* and *Verrucomicrobia* and the metabolites of EPA, acetyllysine, spermine, and others. Endogenous small molecules can be chosen as biomarkers for elucidating the impacts of American ginseng on colitis-related CRC (161). Chen H et al. (21) studied the therapeutic effects of berberine on AOM/DSS-induced CRC in terms of gut microbiota and metabolic changes. Oral berberine significantly reduced colon carcinogenesis by lowering *Actinobacteria* and *Verrucomicrobia* and pathogenic species, increasing some SCFAs-producing bacteria to reinstate microbiota profiles, and regulating glycometabolism, SCFAs metabolism, and amino acid metabolism to reinstate metabolic balance. Red and processed meats are now commonly acknowledged to have a deleterious influence on intestinal homeostasis, as well as pro-inflammatory and dysbiosis-promoting qualities. There is evidence that fortifying pork sausages with inulin had a significant impact on the metabolites produced by the gut microbiome, specifically limiting the formation of undesired N-nitroso compounds in the gastrointestinal tract while enhancing the formation of SCFAs in the colon, thereby preventing people from developing CRC as a result of high red meat consumption (162). According to the findings of the above studies, microbiota and metabolites play a key role in the treatment of CRC by specific medications or diet, which can provide a unique perspective into the inhibitory effects of some drugs on CRC.

## Concluding Remarks and Future Perspectives

This review gives an overview of the changes in the gut microbiome and its metabolites associated with CRC, as well as the application of metabolomics and gut microbiome association analyses in the diagnosis, prevention, and treatment of CRC. It is now well recognized that microbial metabolites may play a significant role in the connection between gut microbiota and CRC risk (163). The gut microbiota converts dietary or herbal phytochemicals, as well as host-derived bile acids and glycoconjugates, into metabolites that influence either the gut flora population or host cells (164, 165). These diverse microbial metabolites can exhibit tumor-suppressive or carcinogenic effects by a range of pathways, including cell cycle changes and immune effector process regulation, as well as transcriptional and epigenetic modification (166). As a result, the microbiome’s disturbance of the metabolite balance can cause the onset and progression of CRC. Metabolomics and gut microbiome association analyses offer new prospects for developing new clinical applications for CRC diagnosis, prevention, and treatment.

To research host-microbe interactions as well as the etiology of CRC, including discovering new treatment targets and the microbiota that distinguishes diseased intestines from those of healthy persons, an integrated investigation of metabolomics and gut metabolic activity is required. Even though most research has discovered dynamic changes in intestinal microbiota composition, gene abundance, and metabolites throughout the multi-stage development of CRC (9, 151, 152), more study is required to fully comprehend the biological mechanism that goes beyond simple association analyses. Because the data on food or drug intervention is mainly obtained from observational research, much further comprehensive longitudinal prospective analyses on specific metabolites, as well as innovative technical advances, are needed to assess whether intestinal microbial and metabolites directly induce tumorigenesis and causality of medicine action.

Molecular pathological epidemiology (MPE), which is focused on disease heterogeneity and molecular pathological traits, is a new discipline that combines epidemiological and pathological study domains. It was established by Shuji Ogino and colleagues (167). MPE seeks to apply epidemiological research design principles and methods to explore the relationship between diet, lifestyle, environmental and genetic exposure factors, disease occurrence, development, and prognosis in order to better understand the etiology and progression of the complex heterogeneous disease and improve therapeutic and preventive measures for clinical medicine and public health (167–169). MPE theories and approaches were gradually adapted to prospective cohort research, which can minimize potential bias associated with case-case and case-control designs, with the onset of the era of personalized/precision medicine and big data. More extensive biological data resources-based assays could be employed in a prospective cohort MPE study to more reliably predict the connection of exposure factors and disease. MPE studies are currently being employed in oncological research, such as CRC, to determine whether certain exposure factors induce certain alterations in a sick individual. For example, Li et al. (170) publish a molecular pathological epidemiology analysis of 945 CRC patients. The mutation rates for the KRAS and BRAF genes were 36.6 percent and 3.46 percent, respectively. KRAS-mutated cancers were more prevalent in female individuals and were never associated with smoking. BRAF-mutated tumors, on the other hand, showed no distinction in terms of gender or smoking status. Furthermore, tumors with BRAF or KRAS mutations were associated with enhanced serum levels of carbohydrate antigen and carcinoma embryonic antigen, suggesting that the integration of serum biomarkers and molecular mutation status may aid in the more precise risk categorization of CRC patients. In addition, MPE studies have shown that frequent aspirin use is associated with a lower risk of CRC with poor tumor-infiltrating lymphocytes but not with a higher risk of CRC with more intense patterns of tumor-infiltrating lymphocytes, indicating that immune responses in the tumor microenvironment play a key role in the chemopreventive effects of aspirin (171). A more extensive investigation of exposure factors, tumor molecular and immunological signatures is critical for better understanding carcinogenesis and generating prognostic biomarkers and targeted therapies. These MPE studies show that the MPE strategy can help with precision CRC medicine and prevention.

As previously stated, host genetic mutations interact with diet, lifestyle, the microbiome, the immune system, and other environmental exposures in the development of CRC, all of which affect disease pathogenesis. The incorporation of microbiology and metabolomics into the MPE model may help to improve knowledge of the complex interacting effects of environment, immunity, microbiome, and individualized molecular biomarkers in CRC (50, 172, 173). Despite its challenges, MPE has distinct strengths that can provide insights into the pathogenic process and aid in the optimization of individualized prevention and therapy. As a result, in the future, a multidisciplinary crossover study, as well as the incorporation of new technologies, will be crucial in uncovering the intricate interactions that occur both within the microbial community and between the microbiota and the host in CRC patients.

## Author Contributions

All authors listed have made a substantial, direct, and intellectual contribution to the work and approved it for publication.

## Funding

This work was supported by grants from the Key Program of Natural Science Foundation of State (Grant No.81830110, 81861168037, 81430093, 81903847), Natural Science Foundation of Heilongjiang Province (YQ2019H030), Scientific and Technology Development Program of Guangxi (AD18126013), the Ba Gui Scholars program of Guangxi, the Central Government Guides Local Science and Technology Development Fund Projects (ZY21195044) and Heilongjiang Touyan Innovation Team Program.

## Conflict of Interest

The authors declare that the research was conducted in the absence of any commercial or financial relationships that could be construed as a potential conflict of interest.

## Publisher’s Note

All claims expressed in this article are solely those of the authors and do not necessarily represent those of their affiliated organizations, or those of the publisher, the editors and the reviewers. Any product that may be evaluated in this article, or claim that may be made by its manufacturer, is not guaranteed or endorsed by the publisher.
